# Role of Directional Configuration in Deep Brain Stimulation for Essential Tremor: A Single Center Experience

**DOI:** 10.5334/tohm.628

**Published:** 2021-11-10

**Authors:** Venka Veerappan, Shannon Anderson, Delaram Safarpour, Amie L. Hiller

**Affiliations:** 1 Department of Neurology, Oregon Health and Science University, Portland, OR, USA; 2 Department of Medicine (Neurology Division), Prisma Health Upstate, Greenville, SC, USA; 3 VA Portland Health Care System, Portland, OR USA

**Keywords:** Deep brain stimulation, essential tremor, directional configuration

## Abstract

**Background:**

Traditionally, the standard of care for medication refractory essential tremor has been to utilize omnidirectional deep brain stimulation of the ventral intermediate nucleus. The advent of directional stimulation allows for spatial restriction of the stimulation on selected targets without involving the neighboring structures, thereby limiting off-target side effects and improving clinical utility.

**Methods:**

We performed a retrospective review of patients between February 2017 and September 2019 who had received ventral intermediate nucleus deep brain stimulation that allowed for directional programming (specifically Abbott/St. Jude). Initial and final major programming sessions post-operatively (approximately 30- and 90-days post-surgery) were examined to determine frequency and reason for use of directional programming.

**Results:**

A total of 33 total patients were identified. A little over half were males (58%, N = 19), with an average age of 68 years old (SD 9.3) at the time of surgery, and a disease duration of almost 30 years (27.2, SD 19) with a wide range from 2–62 years. After initial programming, over 50% (17 of 33) of patients were using directional configurations. This increased to 85% (28 of 33) at the 90-day programming. Reasons for conversion to directional configuration included avoidance of side effects (specifically, muscle contractions (9/33), paresthesia (5/33), dysarthria (1/33) and gait ataxia (1/33)) or improved tremor control (12/33).

**Discussion:**

Our single-center experience suggests that in the large majority of cases, directional leads were utilized and offered advantages in tremor control or side effect avoidance.

## Introduction

Essential Tremor (ET) is one of the most common neurologic movement disorders, with a prevalence of almost 1% in the general population and between 4–6% in persons aged 65 years or older [1]. This hyperkinetic movement disorder is generally initially managed with pharmacologic treatment options including primidone, propranolol, topiramate, gabapentin, and/or non-pharmacologic interventions such as weighted gloves or utensils [2]. There is limited class 1 evidence on pharmacological treatment, and studies have shown that only about half of patients demonstrate clear improvement in tremor control on pharmacological treatment [3]. Furthermore, there are often adverse effects from these medications that limit their utility.

**Figure 1:**
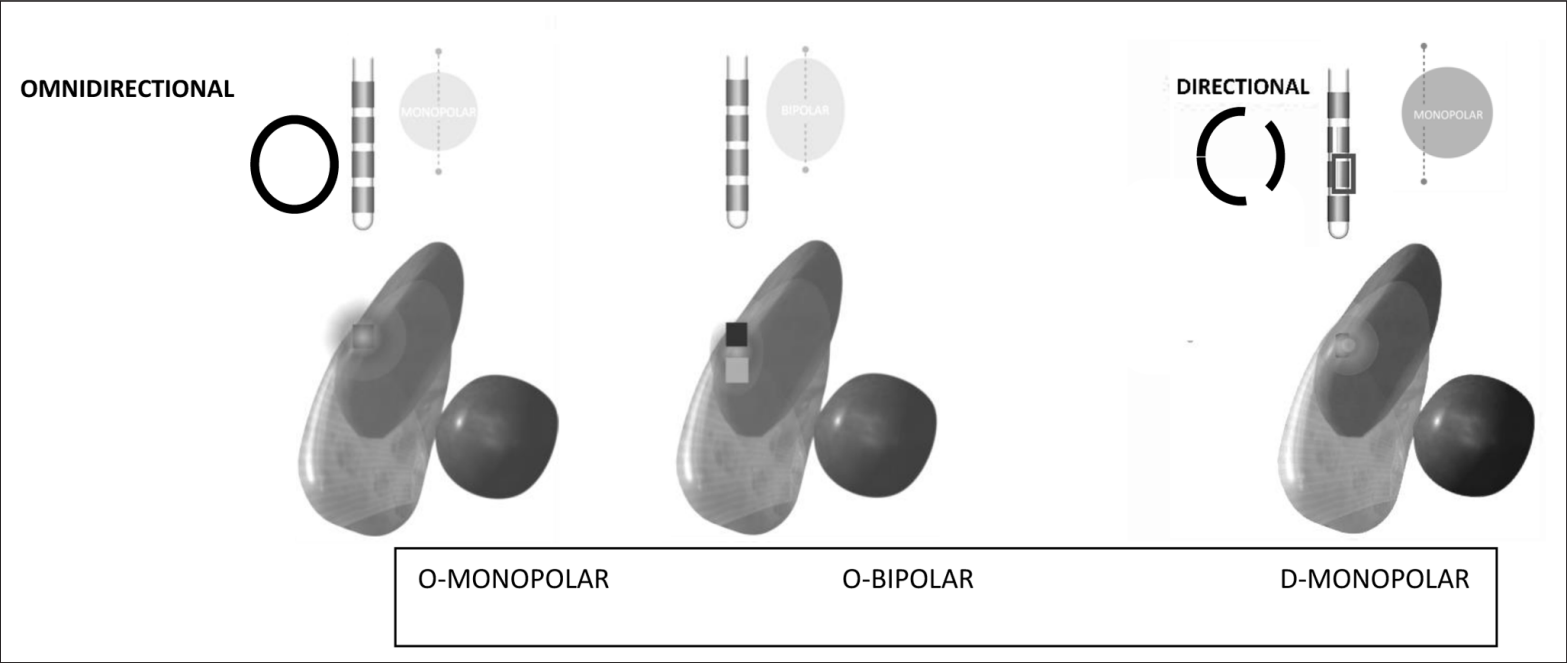
**Omnidirectional and Directional DBS Electrodes**. DBS electrodes generally have four contact points spaced out at variable distances. Omni directional leads are a complete band, and the entire band is either utilized or not utilized. Directional leads on the Abbott/St. Jude system are present on the 2 middle contacts and the band is divided into 3 sections, each of which can be stimulated independently of the others. A bi-polar configuration results in a narrower stimulation field whereas a directional configuration shifts the field laterally. Figure is modified from material provided by Abbott/St. Jude.

Surgical interventions are appealing, especially in patients with medically intractable essential tremor. Prior to the 1990s, the mainstay of surgical treatment for ET had been lesioning of the ventral internal medial (VIM) nucleus of the thalamus. Starting in the 1990’s, led by Benabid et al, deep brain stimulation (DBS) of the thalamic VIM nucleus started to become a much more common surgical treatment for tremor control [4]. Lesional procedures – traditional thalamotomy or focused ultrasound – and VIM DBS tend to reduce tremor amplitude by approximately 90% and are therefore frequently performed in persons with severe tremors [5]. Over the years, DBS technology has advanced, allowing for more options in programming to better manage patients’ symptoms while avoiding side effects [6]. Directional leads have two levels of radially segmented electrodes that afford the ability to restrict the field of stimulation to a more targeted location (see ***Figure 1***). Stimulation of one or more segment(s) of the electrodes can change the shape of the stimulation field to maximize benefits and prevent off-target side effects due to current spread (i.e., paresthesia, muscle contraction, or ataxia) [9]. Research studies have shown that directional stimulation can improve the therapeutic window (the window between minimum stimulation current required to produce side effects and the current required for beneficial effect) and even potentially compensate for small inaccuracies of lead placement [7]. Our center was at the forefront of using this new technology and we performed this retrospective study with the primary goal of determining if this novel DBS technology of directional stimulation is being utilized in a real-world clinical environment and elucidating the reasons. We are aware of one other study looking at the use of directional leads in the clinic done by the Cleveland Clinic and our data is a helpful addition to the body of literature [8]. We feel this information is critical for our own center to understand if directional leads are preferable and for other centers to know if their patients may benefit from using this newer technology.

## Methods

The study was approved by Oregon Health and Science University Institutional Review Board (STUDY 00017680. Deep Brain Stimulation Data Repository). A retrospective electronic chart review was performed using the Oregon Health and Science University Deep Brain Stimulation Clinic Database between February 2017 and September 2019.

At our center, surgical implantation is performed using asleep DBS techniques. For each participant in our review, a preoperative MRI (A 3-D T1-weighted scan (FFE mode, TE 4.60 ms, TR 16.5 ms, GR 30, matrix 256 × 256, 1.3 mm thickness) was obtained and merged for image guided DBS planning on the Medtronic Stealth Station (S7 model hardware, S8 software, version 3.1.1). These were imported into a Stealth Station 7 (Medtronic Corp., Minneapolis, MN, USA). Surgical planning and intraoperative navigation were performed using Frame Link software (version 5.4.1, Medtronic Corp., Minneapolis, MN, USA). For the ventralis intermedius, the second contact on the electrode was targeted at 25% of the distance from posterior commissure (PC) to anterior commissure (AC), 13.5–15 mm lateral to the midline, and at the AC-PC plane. A post-implantation, intraoperative head CT was obtained as part of our standard image-guided DBS implantation protocol [9]. As Abbott was the earliest to bring to market the directional capabilities of segmented leads, this device was chosen as the centerpiece of this study.

**Figure 2:**
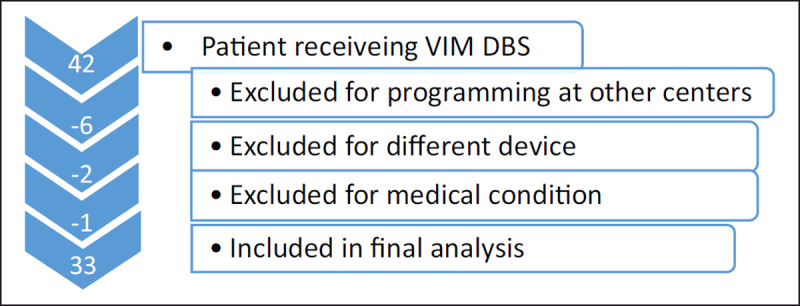
**Patient Enrollment**. Forty-two patients received VIM DBS over the time period we examined. Of those patients the 33 that had the Abbott/St. Jude system, received programming at our center, and were done for essential tremor without significant other neurological disease were included in our analysis.

Our general protocol is to schedule patients for an initial programming visit approximately 30 days after implantation, and then again at 60 and 90 days post-operatively. All patients who had undergone VIM DBS between February 2017 and September 2019 (N = 42) were reviewed. We excluded patients who (1) had not completed all three parts of their initial programming at OHSU (30, 60, 90 days), (2) who had a condition other than ET that was likely to affect the clinical picture (one person excluded for ET/PD syndrome) and (3) who did not have the St. Jude/Abbott device (one person had a Medtronic system and one a Boston Scientific system) (see ***Figure 2***). Key information from the database and on chart review was then extracted including age at time of DBS implantation, sex, number of years since tremor onset, DBS configuration settings at initial programming approximately 30 days from date of surgery – including use of directional versus omnidirectional and the reason for choosing directional current, and follow-up settings at a visit approximately 90 days from date of surgery and again the reason for choosing directional current. Specific tremor rating or quality of life scales were not available as these are not generally done by most programmers and this was a review of clinical practice, not a specific research protocol. The programming strategies varied with different programmers and different patients, as did the degree of details documented in the chart.

## Results

### Patient Characteristics

Following review of the database, a total of 33 patients were identified as having met all inclusion and exclusion criteria for having had surgical implantation of Bilateral VIM DBS for Essential Tremor with Abbott Platform (see ***Table 1***). Of these patients, the sex breakdown was 14 females and 19 males. The mean age at time of surgery was 68 years old (SD 9.3) with disease duration from time of symptom onset being 27 years (SD 18.9).

**Table 1:** Population Demographics. D = Directional and O = omnidirectional.


	TOTAL	AGE (SD)	SEX % MALE (N)	DISEASE DURATION YEARS (SD)	DIRECTIONAL AT 30 DAYS % (N)	DIRECTIONAL AT 90 DAYS % (N)	DIRECTIONAL AT LAST FOLLOW UP % (N)

All Patients	33	68 (9.3)	58% (19)	27.2 (18.9)	51% (17)	84% (28)	88% (29)

D at 90 days	28	68 (9.7)	54% (15)	29.2 (19.0)	57% (17)	100 % (28)	96% (27)

O at 90 days	5	70.2 (4.5)	60 % (3)	16.6 (15.7)	0% (0)	0% (0)	40% (2)

Statistics O vs. D 90-day groups		t(31) = –0.43, *p* = 0.66	Fisher’s Exact χ^2^ = 1.0	t(31) = 1.39, *p* = 0.17	Fisher’s Exact χ^2^ = 0.018		Fisher’s Exact χ^2^ = 0.007


### Data

At time of the 30-day initial programming visit, directional configurations with segmented leads were used unilaterally in 6/33 patients and bilaterally in 11/33 patients, conferring a usage rate of 16.8% and 33.3% respectively. The remaining 16/33 patients were noted to be using traditional omnidirectional settings with ring configuration. All patients in this subgroup using omnidirectional settings were noted to be using the two middle contact levels out of the four possible levels – the two middle contacts can be converted to directional if needed in the future, whereas the top and bottom contact levels are non-segmented and do not have ability to use directional configuration. None of the omnidirectional patients had bipolar configuration at 30 days. Only one patient had a bipolar configuration at this time, and this was in a patient also utilizing a directional configuration. Reasons for use of directional configuration during initial 30-day programming included avoiding stimulation-related adverse effect such as paresthesia (5/17) or muscle contraction (9/17) (see ***Table 2***). The remaining patients (3/17) were configured to use directional settings as they were unable to achieve optimal tremor control as determined by the provider on solely initial omnidirectional settings. We reviewed these three charts in depth to get a sense of process. One chart had very little detail on programming specifics and was complicated by being done only a week after the surgery and patient having some micro-lesion effects. In the other two patients the programmer appears to conduct a mono-polar review of all leads and then a review of all the individual segments of the optimal lead on mono-polar review and noted that tremor control was improved with the directional stimulation.

**Table 2:** Directional Stimulation at 30 and 90 days.


REASON Δ TO DIRECTIONAL	AT 30 DAYS	AT 90 DAYS

Muscle contraction	9	9

Paresthesia	5	5

Tremor control	3	13

Dysarthria	0	1


At 90-day follow up, all patients that were using directional settings (17/33) remained with directional configurations with slight increase in amplitude. Of the 16 patients that were using omnidirectional settings previously at initial 30-day programming visit, 10 were transitioned to directional settings for the purpose of achieving improved tremor control and one to reduce dysarthria. The changeover rate was 68.8%. We reviewed the charts of those where directional settings seemed to provide better tremor control to see what other parameters were adjusted. There was no consistent pattern seen and other changes tried included increase in PW, frequency, and current, as well as bipolar configuration. The remaining 5 patients continued to demonstrate optimal tremor control in omnidirectional settings. Only two patients were transitioned to bi-polar settings at the 90-day programming sessions, one in a directional patient and another in an omni-directional patient. We used a t test and Fisher’s exact test to compare age, sex, and disease duration between those with omni and directional DBS at 30 and 90 days (see ***Table 1***). There were no significant differences between groups in any of these measures. Finally, we examined the most recent follow-up visit for the patients in our cohort. This averaged 24 months (SD 14) from the time of surgery. We found that one patient was transitioned away from a directional configuration and two patients were transitioned from omni-directional to directional settings (see ***Table 1***).

## Discussion

The introduction of segmented leads to the field of deep brain stimulation programming brings the use of directional programming to the forefront. This newer technology has been shown to produce larger VTA in the desired hemi-plane and this is the reason that lower stimulation at a segmented electrode can produce similar effects on tremor control and may lower Total Electrical Energy Delivered (TEED) by the Internal Pulse Generator (IPG) [10]. Current spread beyond the VIM to adjacent pathways can lead to involuntary limb and/or facial muscle contraction from lateral spread into the corticospinal tracts, paresthesia from posterior spread into the sensory ventral caudalis nucleus of the thalamus, and ataxia from medial and posterior spread involving the cerebellar circuitry. Traditionally, the mainstay of programming involved use of ring configurations using non-segmented leads. As such, it was common to encounter unwarranted off-target side effects due to current spread. To minimize adverse effects of stimulation, one had to adjust various programming parameters such as amplitude, pulse width, frequency, and use bi-polar settings. However, the use of directional programming takes advantage of the principal idea that changing shape of stimulation field allows for larger VTA in desired hemi-plane, creating maximal benefits and less adverse effects (see ***Figure 1***). While bi-polar settings can adjust the stimulation field to make it narrower and less likely to cause off target side effects, directional stimulation offers more of a lateral shift of stimulation (see ***Figure 1***).

Abbott (St Jude Medical, St. Paul, MN, USA) was the first DBS platform to develop a directional system; however, later on in 2017, Boston Scientific’s directional Vercise DBS system was FDA-approved based on the results of the INTREPID study [11]. Both of these platforms allow for creation of customized axially-asymmetric directional fields using only a single activated electrode (known as single-segment activation or SSA). When a higher level of customizability of the VTA is required, current fractionalization (distribution of currents through two or more electrodes) can be used. For the Abbott platform, this is possible through interleaving (rapidly alternating two stimulation settings that have different stimulation parameters and a fixed stimulation frequency). For the Boston Scientific platform, current fractionalization can be done through interleaving as well as Multiple Independent Current Control (MICC), which allows for distribution of current in a controlled fashion between contacts [12].

In this single center retrospective study, we demonstrate that the use of directional programming with segmented leads has a strong role to play in the future of managing medication refractory essential tremor. In a group of 33 patients, directional configuration was used in 51.5% of patients at the initial 30-day programming which rose to 84.8% of patients. Fairly similar rates were found in a recent publications from the Cleveland Clinic – 79% of patients utilized directional settings, but they did not indicate why the change was made [8]. In our series, primary reasons for using directional configuration during initial programming was to avoid unwanted off-target adverse effects due to current spread including paresthesia and muscle contractions. In some patients, directional configuration alone offered additional tremor control in the absence of unwarranted adverse effects. Interestingly, patients that were using omnidirectional settings during initial programming were using the middle two contact levels which are segmented as opposed to the top and bottom level which are non-segmented. This takes advantage of the fact that it allows programmers an added level of programming in the future if tremor control remains sub-optimal or patients encounter off-target adverse effects. This was exactly what happened in this small patient population which demonstrated a high conversion rate of 68.8% at time of 90-day programming visit from omnidirectional to directional programming. All of this points to the fact that the anatomy of the VIM nucleus of the thalamus can vary from patient to patient despite normalization with standardized anatomical atlases. Furthermore, it provides an opportunity for novel technologies such as directional leads to help improve overall tremor control and patient outcomes.

We found it interesting and somewhat surprising that so many patients seemed to have directional leads utilized for optimal tremor control. We suspect some of this was related to this being a clinical chart review rather than a research protocol where clinicians might be forced to push stimulation to where side effects are seen and then make choices more based on a therapeutic window. We also wonder if at times side effects where not documented as degree of details varied between individual providers and patients. In informal questioning of our faculty, it was not clear that TEED generally plays a major role in their programming process. Finally, it is interesting how infrequently bi-polar settings were utilized and it appears programmers tended to trial a directional lead prior to trialing a bi-polar configuration.

Some limitations of this study include the retrospective nature of the study, the lack of objective tremor ratings scales such as The Essential Tremor Rating Scale – TETRAS [13] – to have objective data regarding degree of tremor improvement in the “ON” stimulation state, the lack of quality of life measures, and the limited details available from some programmers. Finally, the study size was limited but with this being a newer technology, large numbers of patients have not yet been implanted with this device. Hopefully, this can be expanded for future studies as needed.

Overall, in our experience, this retrospective review demonstrates that the use of directional stimulation with segmented leads appears to confer additional benefits to both patients and DBS programmers over traditional omnidirectional configurations for improved tremor control and avoidance of unwanted off-target adverse effects.
